# Self-Rated Health and Living Status Across Different Ages at Later Life: A Population Study in Hong Kong

**DOI:** 10.1093/geroni/igaf122.2464

**Published:** 2025-12-31

**Authors:** Jasen Kin-Fung Leung, Eliza Lai-Yi Wong

**Affiliations:** The Chinese University of Hong Kong, Hong Kong, China (People’s Republic); The Chinese University of Hong Kong, Hong Kong, China (People’s Republic)

## Abstract

There is growing attention to the relationship of self-rated health (SRH) with living arrangements, especially when issues like social isolation are brought into the limelight, necessitating urgent social policy interventions. This study is a part of a population-level biannual patient experience survey conducted in Hong Kong to study the relationship between SRH and living arrangements across older age groups. Hong Kong residents, aged 18 or above, who stayed in a public hospital for at least a night, were contactable within two weeks after discharge, and were able to communicate in either Cantonese, English, or Mandarin, were eligible. A total of 32,562 participants, aged 51 to 109, were included from five waves of surveys between 2013 and 2023. Despite the mean of SRH scores peaking at 3.27 in 2019, it experienced a significant decline in 2023, reaching 3.03, a potential pandemic aftermath. Delving into SRH and living status, ANOVA results showed significant differences in SRH among older adults of different living arrangements. Tukey’s HSD tests showed older adults aged 51-60 (means differences = -4.42, p-value = 0.00), 61-70 (means differences = -1.98, p-value = 0.00), and 71-80 (means differences = -1.39, p-value = 0.07), reported a significantly better SRH when they live with others; whilst among those who aged 81 or above, living alone is significantly associated with a better SRH (means differences = +2.62, p-value = 0.00). Future research could investigate the mechanisms underlying such discrepancies, for instance, how living and caregiving arrangements constitute older adults’ subjective well-being.

